# 

**Published:** 1890-02-08

**Authors:** 


SATURDAY, FEBRUARY 8th, 1890.

**Is Vaccination a Fraud?** - 287

**Words of Consolation.**—"Unsatisfied" - 288

**Village Charities.**—I. Hard and Fast Rules of Distribution - 289

**The Christian Origin of Hospitals** - 290

**The Doctor's Pulpit.**—A Plea for the Disappointed - 291

**The History and Capabilities of Herbal Simples.**—IV. The Apple. By W. T. Fernie, M.D. - 292

**Everybody's Page** - 293

**News and Notes** - 294

**Annotations.**—Unpardonable—Important to Charities—Pauper Lunatics in Licensed Houses—The Last of the Influenza - 296

**Fallow Crops.**—A Kirmes at Buffalo - 302

**Scraps and Gleanings** - 302

**The Practitioner's Outlook and Retrospect:**
Medicine—Compression of the Thorax for Relieving the Pain of Pleurisy—Cure of Leprosy—Mental Blindness—The Temperature in Influenza - 297
Surgery—Tracheotomy in Diphtheria—Silk as a Dressing 298
Hygiene—Milk Poisoning - 298
Recent Research—Injury to the Phrenic Nerve - 299
Dietetics—Meat Diet - 299
Therapeutics—Acetanilide—False Croup - 299
New Drugs, Appliances, and Things Medical—Gunn's "Food of Life"—Alexander's Beef Wine - 300
**Turning Over New Leaves.**—"Father Damien"—"A More Excellent Way"—"Chats at St. Ampelio"—"Ralph Ellison's Opportunity"—"Subjects of Social Welfare" - 300

### EXTRA SUPPLEMENT—THE NURSING MIRROR.

**En Passant.**—The Midwives' Institute—The "Queen"—Cornwall Nurses' Home—Thousands of Pounds for the National Pension Fund—The Brassey Holiday Home—In Aid of an Institute—Short Items—Rural Nursing Association - lxxvii
**Introductory Lecture** to the Course Given at the Royal Hants Infirmary, Southampton. By Percy G. Lewis, M.D. - lxxviii
**Winchester Training School for Nurses** - lxxviii
**Examination Questions** - lxxix
**Everybody's Opinion** - lxxix
**Death in Our Ranks** - lxxix
**Exhibition of Nursing Uniforms** - lxxix
**Appointments—Notes and Queries—Vacancies—Amusements** - lxxx

